# Long‐term effectiveness and cost‐effectiveness of collaborative dementia care: A general practitioner based randomized controlled trial

**DOI:** 10.1002/alz.088810

**Published:** 2025-01-09

**Authors:** Bernhard Michalowsky, Iris Blotenberg, Moritz Platen, Ingo Kilimann, Stefan Teipel, Jochen René Thyrian, Wolfgang Hoffmann

**Affiliations:** ^1^ Deutsches Zentrum für Neurodegenerative Erkrankungen e. V. (DZNE), site Rostock / Greifswald, Greifswald Germany; ^2^ German Center for Neurodegenerative Diseases e.V. (DZNE), Site Rostock/Greifswald, Greifswald Germany; ^3^ Department of Psychosomatic Medicine, Rostock University Medical Center, Rostock Germany; ^4^ German Center for Neurodegenerative Diseases, Rostock Germany; ^5^ University of Rostock, Rostock Germany; ^6^ Deutsches Zentrum für Neurodegenerative Erkrankungen e.V. (DZNE) and Institute for Community Medicine / University of Greifswald, Greifswald Germany; ^7^ Institute for Community Medicine / University of Greifswald, Greifswald Germany; ^8^ Institut for Community Medicine, University Medicine, University of Greifswald, Greifswald Germany

## Abstract

**Background:**

Previous trials reported that collaborative Dementia Care Management (cDCM) could be effective for patients and caregivers and cost‐effective for healthcare systems in the short term. However, long‐term evidence is lacking. Therefore, the study’s objective was to determine the long‐term efficacy and cost‐effectiveness of cDCM compared with usual care.

**Method:**

A General Practitioner (GP)‐based, cluster‐randomized‐controlled intervention trial (DelpHi‐MV) was conducted. Participating GP practices were randomly allocated to one of two arms (care as usual or cDCM). Participants of the intervention group received a comprehensive needs assessment and individualized interventions by nurses specifically qualified for dementia collaborating with GPs and healthcare stakeholders over six months. Controls received usual care. Primary endpoints were behavioral and psychological symptoms (NPI), caregiver burden (Berlin Inventory of Caregivers' Burden), Health‐Related Quality of Life (HRQoL, QoL‐AD, SF‐12), anti‐dementia drug treatment, potentially inappropriate medication, and cost‐effectiveness (incremental cost and Quality‐adjusted Life Years, QALYs) after 36 months.

**Result:**

308 participants (n = 221 cDCM, n = 87 usual care) were included for the efficacy analyses, and 428 (n = 303 cDCM, n = 125 usual care) for the cost‐effectiveness analysis that included deceased patients. Based on multivariate regression models adjusted for baseline scores, participants receiving cDCM showed significantly less behavioural and psychological symptoms (adjusted mean difference ‐10.3 [95% CI ‐16.9 to ‐3.6], p = 0.003, Cohens‐d = ‐0.78), better mental health (+2.26 [0.3 to 4.2], p = 0.023, d = 0.26) and lower caregiver burden (‐0.59 [‐0.8 to ‐0.4], p<0.001, d = 0.71), and more likely received anti‐dementia drugs (adjusted odds ratio 1.91 [1.0 to 3.8], p = 0.065, Cramérs‐V = 0.12) compared to usual care participants. There was no effect on overall HRQoL, physical health, or potentially inappropriate medication after 36 months. cDCM gained QALYs (+0.14 [0.01 to 0.27], p = 0.050, d = 0.20) and increased costs (+437€ [‐5,438 to 6,313], p = 0.868, d = 0.02), resulting in a cost‐effectiveness‐ratio of 3,186€/QALY. Cost‐effectiveness was significantly better in PlwD living alone (‐3,815€, +0.224 QALYs, cDCM dominates) compared to those living with a caregiver (+3.283€, +0.079 QALYs, 47,538€/QALY).

**Conclusion:**

cDCM is effective and cost‐effective in the long term, improving patients, caregivers, and health system‐relevant outcomes beyond the intervention or short‐term periods, and, therefore, should become a health policy priority and translated into routine care practice.

